# Molecular interactions of *Toxoplasma gondii* dense granule 23 (GRA23) with host proteins PEX3 and TRAP1

**DOI:** 10.3389/fvets.2025.1585261

**Published:** 2025-05-09

**Authors:** Xuewei Fan, Nan Zhang, Xichen Zhang, Shudong Li, Jiangming Fu, Min Sun, Xiaoxiao Ma, Muhammad Zahoor Khan, Xin Jiang

**Affiliations:** ^1^College of Veterinary Medicine, Jilin University, Changchun, Jilin, China; ^2^Heilongjiang Agricultural Economy Vocational College, Mudanjiang, China; ^3^College of Agriculture and Biology, Liaocheng University, Liaocheng, China

**Keywords:** *Toxoplasma gondii*, GRA23, BiFC, PEX3, TRAP1

## Abstract

**Background:**

*Toxoplasma gondii*, an obligate intracellular protozoan, utilizes dense granule proteins to modulate host cell processes. Dense granule protein 23 (GRA23) facilitates molecular trafficking between the parasitophorous vacuole and host cell cyto-plasm, though its specific host protein interactions remain poorly characterized.

**Methods:**

This study employed pull-down assays coupled with mass spectrometry to identify host proteins interacting with GRA23.

**Results:**

Among 35 proteins identified, peroxisomal biogenesis factor 3 (PEX3) and TNF receptor-associated protein 1 (TRAP1) were validated through bimolecular fluorescence complementation (BiFC) and co-immunoprecipitation (Co-IP) assays. Gene Ontology (GO) and Kyoto Encyclopedia of Genes and Genomes (KEGG) analyses revealed significant enrichment of these interacting proteins in metabolic pathways and cellular processes related to reproduction, growth, and development.

**Conclusion:**

The interaction between GRA23 and PEX3 suggests potential parasite modulation of peroxisomal functions, while its association with TRAP1 indicates possible exploitation of host chaperone mechanisms. This study provides the first evidence that GRA23 interacts with host proteins implicated in key cellular functions, offering novel insights into *T. gondii* pathogenesis and potential therapeutic targets for toxoplasmosis treatment.

## 1 Introduction

*Toxoplasma gondii* is a globally widespread opportunistic protozoan parasite capable of infecting virtually all warm-blooded vertebrates, including humans (1). Approximately 30% of the global human population is infected with *T. gondii*, primarily through ingestion of food and water contaminated with sporulated oocysts shed by infected cats, consumption of undercooked or raw meat containing tissue cysts, or via congenital transmission from infected mothers to their fetuses (2–4). *T. gondii* infection has significant veterinary (5–7) and public health implications, often remaining asymptomatic in immunocompetent individuals but causing severe disease in those with compromised immune systems, such as HIV-infected patients and other immunocompromised individuals (8). In pregnant women, primary infection with *T. gondii* can result in tachyzoite invasion across the placental barrier, potentially leading to spontaneous abortion or congenital birth defects (9). The parasite's broad host range is attributed to its effective mechanisms for host invasion and immune modulation.

Within the cytoplasm of *T. gondii*, three types of secretory organelles are present: micronemes, rhoptries, and dense granules. Micronemes and rhoptries release their contents from the apical pole of the parasite, while dense granules discharge from the apical, lateral, and posterior regions (10, 11). The release of these organelles' contents follows a well-defined cascade. Upon contact with host cells, micronemes are involved in the trafficking and sequestration of binding ligands for host cell receptors, and ensure the appropriate release of these ligands in high concentration at the very tip of the parasite, in response to external stimuli that sense contact with the host cell surface (12, 13). Subsequently, the contents of the rhoptries (ROP1-ROP9) are secreted, rhoptries are unique secretory organelles shared by all Apicomplexan invasive stages. They are exocytosis upon host cell invasion and their contents are involved in creating the moving junction that propels the parasite in the cell and in building the parasitophorous vacuole (PV) in which the parasite will develop (14). The exocytosis of dense granule proteins occurs both during and after parasite invasion into the PV (15). These proteins either remain soluble within the PV lumen or associate with the PV membrane and the tubulo-reticular membrane network within the PV. It is believed that these GRA proteins modify the PV environment, contributing to the parasite's intracellular survival and replication. Notably, GRA proteins are constitutively released in a calcium-independent manner prior to host cell entry and form a significant portion of the excretory/secretory proteins (ESP) of *T. gondii*.

Several GRA proteins in *T. gondii* have been extensively studied for their roles in various cellular processes. For instance, GRA2, GRA4, GRA6, and GRA12 are involved in constructing the intravacuolar network, enabling interparasite connectivity within the parasitophorous vacuole and bridging them to the parasitophorous vacuole membrane (PVM) (16, 17). GRA7 facilitates the formation of tubulo-structures sequestering host organelles at the PVM, and GRA15, a polymorphic effector, activates the NF-κB pathway (18, 19). GRA16, 18, and 24 extend modulating host gene expression (20–22). GRA22 and GRA41 are crucial for parasite egress, whereas GRA25, mainly stimulates the expression of chemokines (Such as CCL2 and CXCL1) (23–25).

Recently, GRA23 has been identified as a secretory protein in *T. gondii* that facilitates the transfer of small molecules between the PV and the host cell cytoplasm, providing a molecular basis for how the parasite acquires nutrients from host cells (26). However, the specific host proteins interacting with GRA23 remain unidentified. Therefore, we designed this study to identify these interacting proteins, aiming to provide deeper insights into the functional mechanisms of GRA23 and enhancing our under-standing of the interplay between *T. gondii* and its host proteins.

## 2 Materials and methods

### 2.1 Ethical statement

All animal experiments were reviewed and approved by Animal Welfare and Research Ethics Committee at Jilin University (IACUC Permit No. SY202407014).

### 2.2 Parasite, cell culture and antibodies

The serial propagation of tachyzoites from the *T. gondii* RH strain was conducted in monolayers of human foreskin fibroblasts (HFFs). HFFs were maintained in Dulbecco's Modified Eagle's Medium (DMEM) supplemented with 10% fetal bovine serum (FBS), 2 mM glutamine, 100 μg/mL streptomycin, and 100 U/mL penicillin in a humidified incubator at 37°C with 5% CO_2_. The HEK293T cell line was cultured in RPMI 1640 medium (Hyclone, USA) supplemented with 10% FBS (Gibco, USA) and 1% penicillin/streptomycin (Gibco, USA). The antibodies used in this study included anti-HA tag (Sigma-Aldrich), anti-GST tag (Abcam), and rabbit polyclonal antibody against recombinant GRA23 (prepared as described below).

### 2.3 Production of recombinant GST-tagged GRA23 protein

Total RNA was extracted using an Ambion RNA kit and reverse transcribed into cDNA using random hexamers with the SuperScript™ II kit (Invitrogen) according to manufacturer's protocol. PCR primers were designed (Supplementary Table S1) to amplify the GRA23 gene using *T. gondii* genomic DNA as template. The amplicons were digested with *Sma*I and *Not*I restriction enzymes and purified by agarose gel electrophoresis. The purified DNA was ligated into the pET-41a expression vector (pre-digested with the corresponding enzymes) using T4 DNA ligase at 16°C overnight. The constructed plasmids were extracted and verified by sequencing. The successfully constructed expression plasmid was designated pET-41a-GRA23.

For protein expression, pET-41a-GRA23 was transformed into BL21 (DE3) competent cells, with the empty pET-41a vector serving as negative control and non-transformed cells as blank. Protein expression was induced with 1 mM Isopropyl b-D-1-thiogalactopyranoside (IPTG) at 16°C for 8 h. The bacterial cells were harvested by centrifugation, lysed by sonication, and the expressed proteins were analyzed by SDS-PAGE. The recombinant GRA23-GST fusion protein was purified using glutathione-agarose affinity chromatography. The purified protein was validated by Western blot analysis using anti-GRA23 antibody.

### 2.4 Preparation of GRA23 polyclonal antibody

To generate polyclonal antibodies against GRA23, purified recombinant GRA23-GST fusion protein (immunogen) was mixed with Freund's complete adjuvant (1:1 ratio) and thoroughly emulsified. New Zealand white rabbits were immunized subcutaneously with 400 μg of protein per rabbit at multiple sites. For booster immunizations, the GRA23 protein was mixed with Freund's incomplete adjuvant (1:1 ratio), emulsified, and administered weekly for three consecutive weeks. One week after the final immunization, blood was collected by cardiac puncture to obtain immune serum, which was stored at −80°C. The specificity of the anti-GRA23 polyclonal antibody was confirmed by Western blot analysis using *T. gondii* whole protein lysate, with goat anti-rabbit antibody (Thermo Fisher) as the secondary antibody.

### 2.5 GST pull-down and LC-MS/MS analysis

Recombinant GRA23-GST protein was immobilized onto Glutathione Sepharose 4B resin and equilibrated with GST binding buffer. Vero cell lysate was used to extract total proteins, and then incubated with the immobilized GRA23-GST fusion protein or GST alone (control) at 4°C for 2 h with gentle rocking. After incubation, the resin was washed thoroughly with GST binding buffer to remove non-specifically bound proteins. Bound proteins were eluted using reduced glutathione solution and stored at −20°C for further analysis. The eluted proteins were separated by SDS-PAGE. Protein bands unique to the GRA23-GST pull-down (compared to GST control) were excised and submitted to Shanghai Bio-profile Biotechnology Co., Ltd. for identification by liquid chromatography-tandem mass spectrometry (LC-MS/MS).

### 2.6 Gene ontology /Kyoto Encyclopedia of Genes and Genomes analysis

Mass spectrometry data were analyzed using ProteinPilot software to identify proteins, followed by Wayne diagram analysis. After subtracting proteins identified in the control group, the remaining proteins from the experimental group were subjected to Gene Ontology (GO) annotation to elucidate their molecular functions, cellular components, and biological processes. Additionally, the Clusters of Orthologous Groups (COG) database was used to functionally classify the identified proteins, while potential signaling pathways were explored using the KEGG database. Subcellular localization predictions were performed using WoLF-PSORT (https://wolfpsort.hgc.jp/) and PSORTb (https://psort.org/documentation/index.html).

### 2.7 Co-immunoprecipitation

Based on the sequences of identified interacting proteins PEX3 and TRAP1, primers containing BamHI and XhoI restriction sites were designed to amplify these genes by PCR (Table 1). The PCR products were verified on 1% agarose gel. The pcDNA3.1-HA vector was digested with *Bam*HI and *Xho*I, and the PEX3 and TRAP1 gene fragments were ligated into the linearized vector. The recombinant plasmids pcDNA3.1-HA-PEX3 and pcDNA3.1-HA-TRAP1 were obtained following transformation into competent cells.

**Table 1:** The primers used in the present study.

**Primers**	**Length**	**Sequence(5^′^-3^′^)**
GRA23-F	22	GACGACGCCTTCATAGACAATG
GRA23-R	22	CTAGTTCTTTCGCGCAAGGGGT
PEX3-F	22	ATGCTGAGGTCCGTATGGAATT
PEX3-R	19	TCATTTCTCCAGTTGCTGA
TRAP1-F	21	ATGGCGCGCGAGTTGCGGGCG
TRAP1-R	20	TCAGTGTCGCTCCAGGGCCT

To investigate the interactions between GRA23 and the candidate proteins, pcDNA3.1-GST-GRA23/pcDNA3.1-HA-PEX3 and pcDNA3.1-GST-GRA23/pcDNA3.1-HA-TRAP1 were co-transfected into Vero cells separately. After 24 h, cells were washed three times with PBS and lysed at 4°C for 30 min. Cell lysates were incubated with either anti-GST or anti-HA magnetic beads on a rotary shaker at 4°C overnight. The beads were then washed three times with lysis buffer, boiled in SDS loading buffer at 100°C for 10 min, and the eluted proteins were analyzed by Western blot.

### 2.8 Bimolecular fluorescence complementation assay

The coding sequences of GRA23, PEX3, and TRAP1 were obtained from NCBI, and restriction enzyme sites were identified for the pBiFC-VC155 and pBiFC-VN173 vectors. Using seamless cloning technology, the GRA23 gene was inserted into the pBiFC-VC155 vector, while PEX3 and TRAP1 genes were inserted into the pBiFC-VN173 vector, creating recombinant plasmids pBiFC-GRA23-VC155, pBiFC-PEX3-VN173, and pBiFC-TRAP1-VN173, respectively. To verify protein-protein interactions, endotoxin-free plasmid pairs (pBiFC-bfos-VC155/pBiFC-bjun-VN173 as positive control, pBiFC-VC155/pBiFC-VN173 as negative control, pBiFC-GRA23-VC155/pBiFC-PEX3-VN173, and pBiFC-GRA23-VC155/pBiFC-TRAP1-VN173) were transfected into Vero cells using Lipofectamine 2,000 (Invitrogen, Carlsbad, California, USA) transfection reagent. After 24 h, cells were fixed with 4% paraformaldehyde, counterstained with 4′, 6-diamidino-2-phenylindole (DAPI; Biotium, Fremont, CA, USA) to visualize nuclei, and examined under a fluorescence microscope.

## 3 Results

### 3.1 Production of a recombinant GST-tagged bait protein GRA23

**Figure 1:**
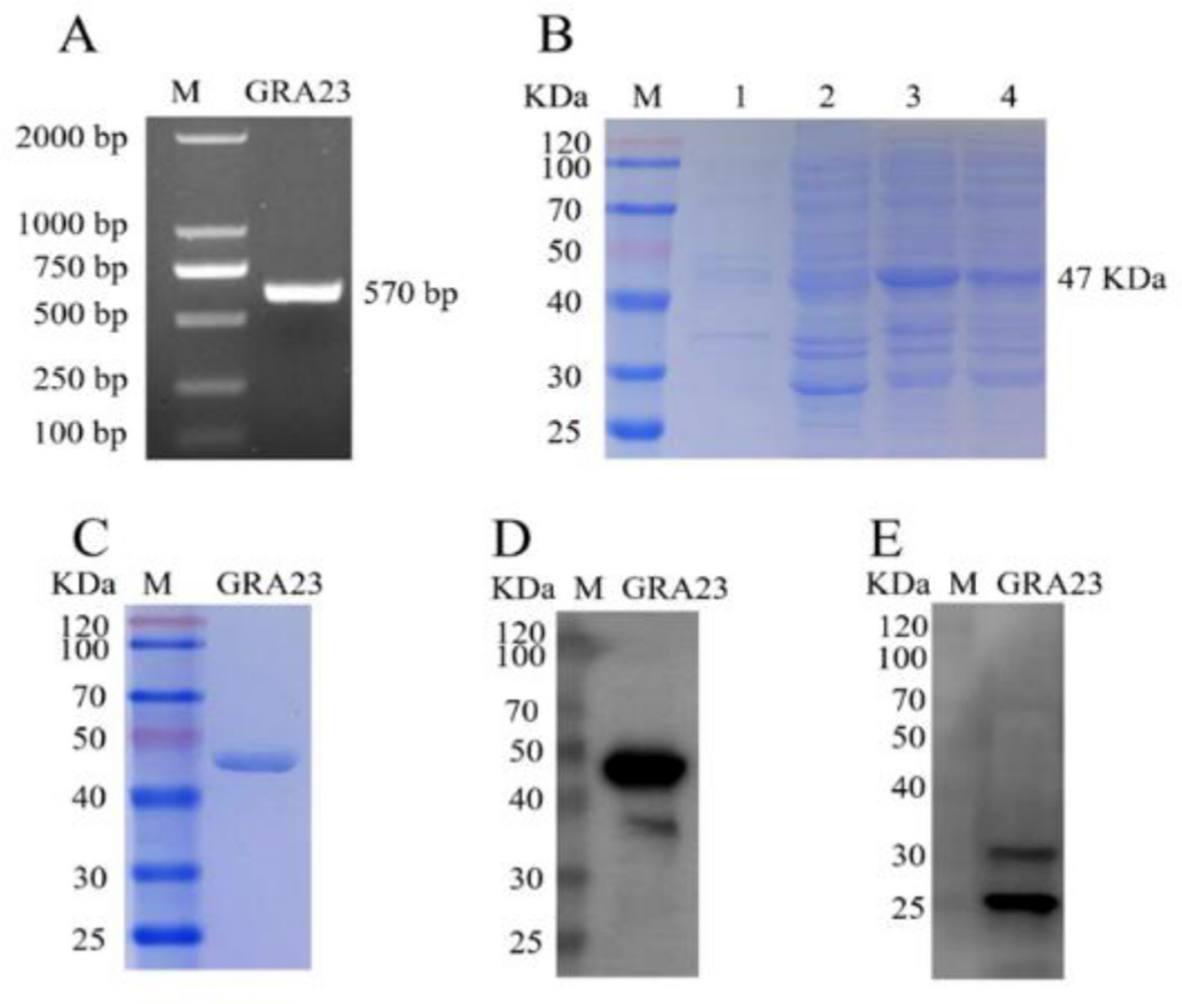
Cloning, expression, and identification of GRA23. **(A)** Amplification of target genes. **(B)** Induced expression of target protein. 1–2: supernatant and precipitation of pET41a empty vector; 3–4: supernatant and precipitation of pET41a-GRA23 vector. **(C)** Purification of target protein. **(D)** Identification of purified protein using GST monoclonal antibody. **(E)** Identification of GRA23 protein using immunoblotting.

Using the total cDNA of *T. gondii* as a template, the full-length fragment of the GRA23 gene was amplified by PCR. Agarose gel electrophoresis revealed a band of ~570 bp, which was consistent with the expected size (Figure 1A). To further characterize this protein, the gene was cloned into the prokaryotic expression vector pcDNA3.1, resulting in the successful construction of the recombinant plasmid pET-41a-GRA23, which was then transformed into BL21 cells capable of expression. SDS-PAGE results showed that the GRA23 protein was induced in both the supernatant and precipitate at 16°C (Figure 1B). Further validation through immunoblotting using an anti-GST tag antibody as the primary antibody revealed a band at 47 kDa, indicating that the protein was purified (Figures 1C, D). Subsequently, rabbit polyclonal antisera against GRA23 were prepared, and immunoblotting analysis was conducted on the recombinant protein to identify the native protein expression in *T. gondii*. Western blot analysis of *T. gondii* total proteins was performed using rabbit serum immunized with GRA23 protein as the primary antibody and goat anti-rabbit antibody as the secondary antibody. The results demonstrated the presence of a band at 24 kDa, confirming the existence of the GRA23 protein in *T. gondii* (Figure 1E).

### 3.2 GST pull-down and LC-MS/MS analysis

We utilized *Escherichia coli* to express the GST-fused GRA23 protein. After purification, GST pull-down was performed, where Vero cell lysate was incubated with glutathione agarose conjugates carrying either GST or GST-GRA23 protein, followed by elution to capture interacting proteins from the Vero cell lysate. SDS-PAGE electrophoresis was conducted, followed by Coomassie brilliant blue staining and destaining with destaining solution, revealing two groups of bands with significant differences. LC-MS/MS was employed to identify the proteins interacting between GRA23 and the host proteins from Vero cells. Proteins identified in the GST-only control group were excluded, initially screening out 35 proteins potentially interacting with GRA23 (Supplementary Table S1).

### 3.3 GO/KEGG analysis

**Figure 2:**
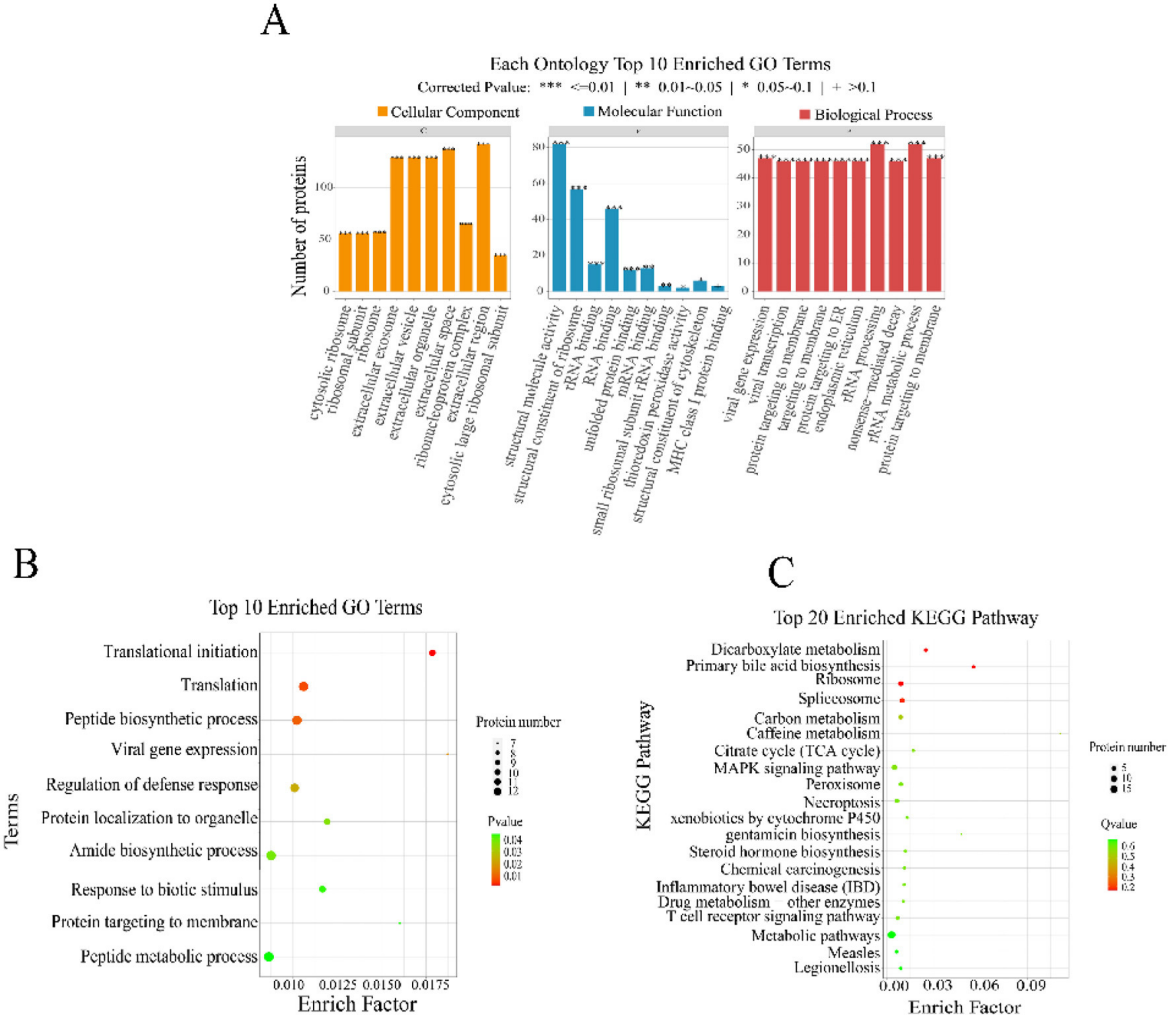
KEGG and GO analysis of the obtained CO-IP proteins. **(A)** Each ontology top 10 enriched GO terms. **(B)** Top 10 enriched GO terms. **(C)** Top 20 enriched KEGG pathway.

To analyze the function of the screened proteins, we utilized the GO database and the KEGG database to annotate the differential genes in terms of molecular function, biological process, cellular component, and signaling pathways. Concurrently, we applied protein enrichment algorithms to filter and select significantly enriched GO terms and signaling pathways. All differential proteins were mapped to all GO terms and signaling pathways in the KEGG database, and the number of differentially expressed proteins in each GO term or KEGG pathway was calculated. GO functional analysis was conducted on the initially screened 35 proteins that potential interacts with Vero cells. These interacting proteins cover cellular structures, intracellular substances, and protein containing complexes (Figure 2A). They are involved in biological processes such as metabolism, cellular metabolism, and biological regulation, and possess binding activity, catalytic activity, and molecular function regulatory activity (Figure 2B). Particularly, these proteins are significantly enriched in processes like translation initiation, translation, and peptide biosynthetic process. KEGG analysis indicated that these proteins are also involved in signal transduction pathways related to human diseases, cellular processes, bodily systems, and metabolism (Figure 2C).

### 3.4 Co-IP validation of GRA23 interactions

**Figure 3:**
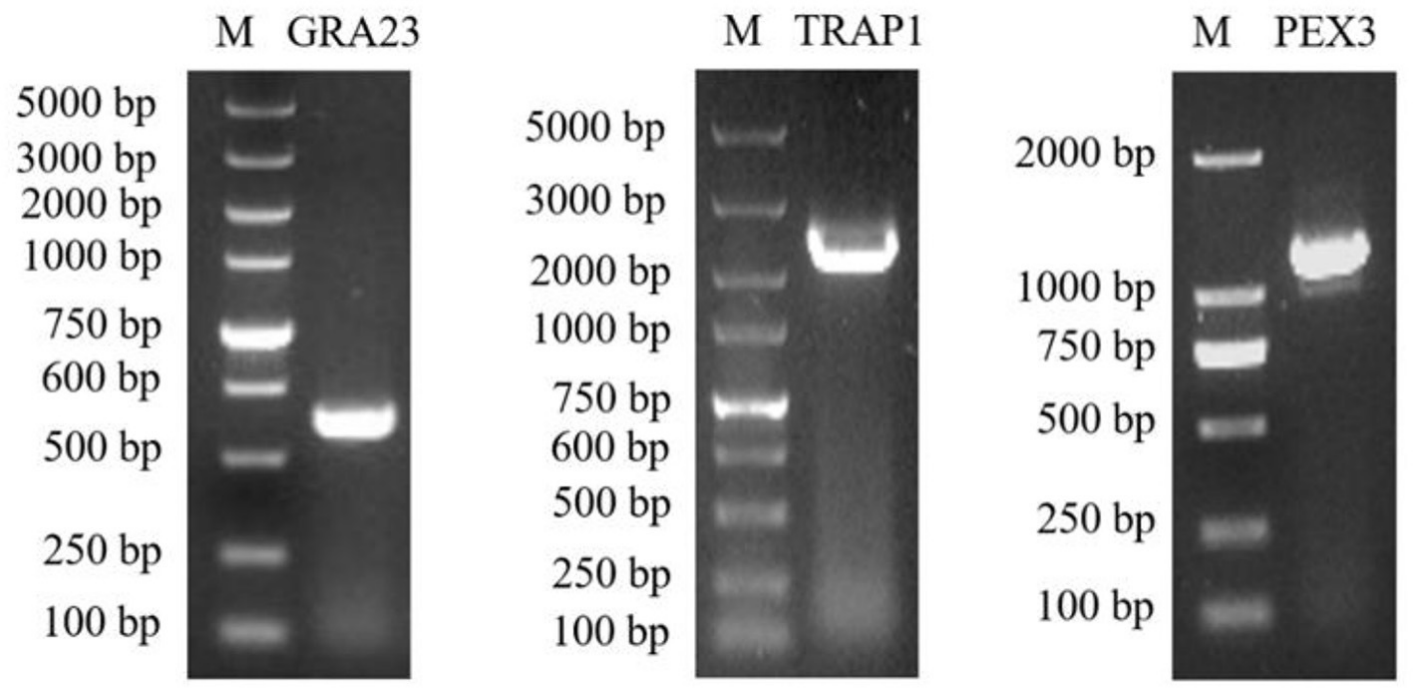
The PCR amplification of target GRA23, TRAP1, and PEX3 genes.

The acquisition of recombinant plasmids pcDNA3.1-HA-PEX3 and pcDNA3.1-HA-TRAP1 has been accomplished, with PCR outcomes aligning with our expectations (Figure 3). For the co-transfection assays, proteins from the pcDNA3.1-GST-GRA23/pcDNA3.1-HA-PEX3 group were interacted with HA magnetic beads and the same procedure was applied to the pcDNA3.1-GST-GRA23/pcDNA3.1-HA-TRAP1 group. The lysate of the pcDNA3.1-GST-GRA23-transfected group was treated with GST magnetic beads, whereas the pcDNA3.1-HA-PEX3 and pcDNA3.1-HA-TRAP1 groups were each interacted with HA magnetic beads.

**Figure 4:**
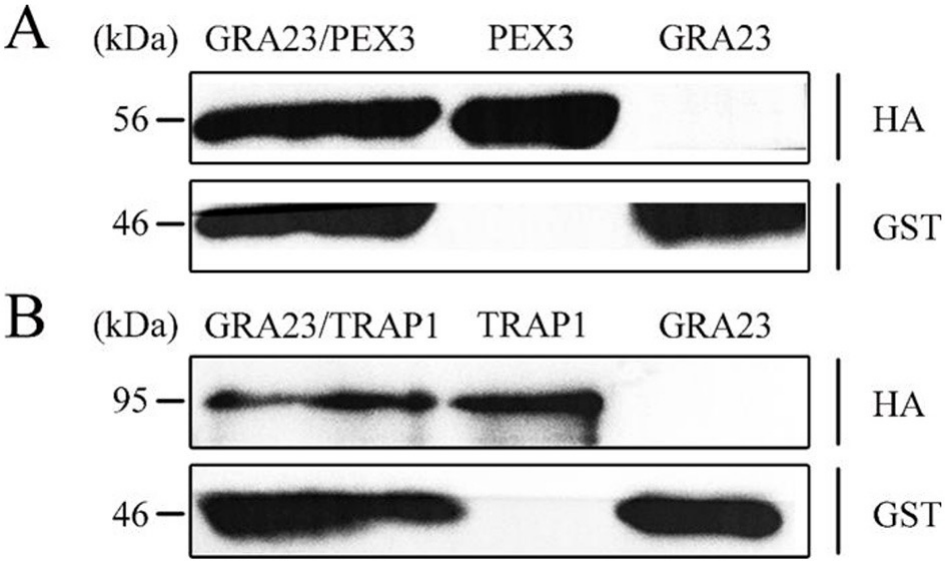
Validation of interacting proteins. **(A)** Verification of interaction between GRA23 and PEX3 by Western blotting. **(B)** Verification of interaction between GRA23 and TRAP1 by Western blotting.

Our findings revealed that the pcDNA3.1-GST-GRA23/pcDNA3.1-HA-PEX3 co-transfection group produced GST-GRA23 (47 kDa) and HA-PEX3 (56 kDa) proteins (Figure 4A), and the pcDNA3.1-GST-GRA23/pcDNA3.1-HA-TRAP1 group produced GST-GRA23 and HA-TRAP1 (96 kDa) proteins (Figure 4B). In contrast, individual transfection groups for pcDNA3.1-GST-GRA23, pcDNA3.1-HA-PEX3, and pcDNA3.1-HA-TRAP1 detected only the respective expression of GST-GRA23, HA-PEX3, and HA-TRAP1 proteins. These results indicate the presence of interaction motifs within GRA23 that engage with PEX3 and TRAP1, underscoring a significant interactive association.

### 3.5 BiFC validation of GRA23 interactions

**Figure 5:**
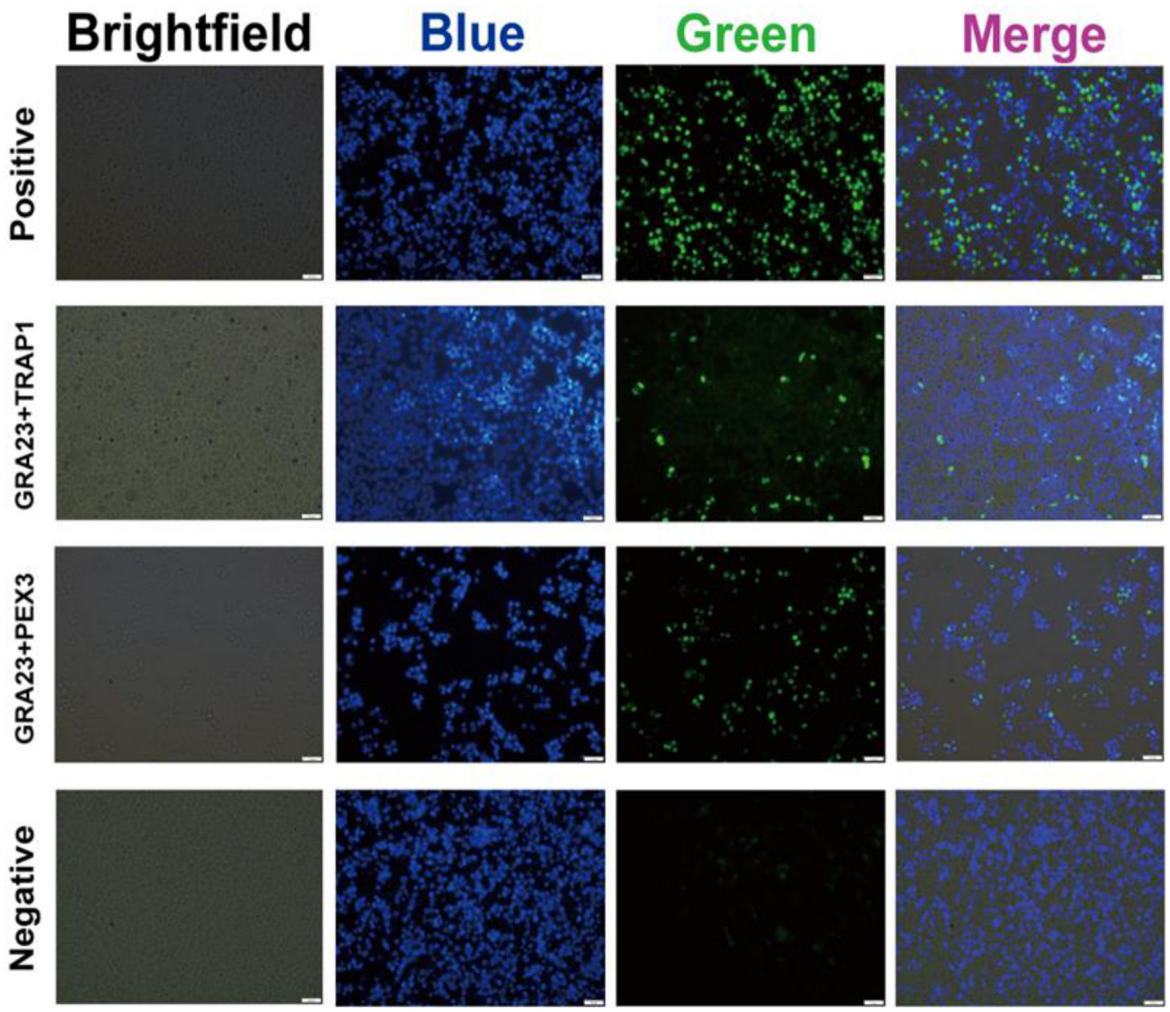
Bimolecular fluorescence complementary of GRA23, PEX3, and TRAP1.

In order to ascertain the interaction of GRA23 with PEX3 and TRAP1 in Vero cells, we performed a BiFC experiment. The findings revealed green fluorescence signals in the groups co-transfected with pBiFC-bfos-VC155/pBiFC-bjun-VN173, pBiFC-GRA23-VC155/pBiFC-TRAP1-VN173, and pBiFC-GRA23-VC155/pBiFC-PEX3-VN173. However, no green fluorescence was detected in the negative control group with pBiFC-VC155/pBiFC-VN173. This discovery suggests that GRA23 directly engages with PEX3 and TRAP1 within Vero cell (Figure 5).

## 4 Discussion

*T. gondii*, an extensively distributed opportunistic pathogen, infects nearly all warm-blooded animals, including humans (3). Its intricate pathogenicity hinges significantly on the interactions with host cells (27). Recent advancements in molecular biology and proteomics have substantially facilitated profound investigations into the *T. gondii*-host cell dynamics (28). GRA23, as an integral component of the dense granule protein family, potentially modulates host cell bioactive factors, thereby impacting metabolic pathways and immune responses, which consequently may promote *T. gondii*'s intracellular invasion, parasitism, and proliferation (29–31). This study expands on earlier research by Gold et al. (26) by identifying GRA23-interacting proteins using proteomics and biochemical approaches. The current study employs robust experimental techniques, including pull-down assays, mass spectrometry, co-immunoprecipitation (Co-IP), and bimolecular fluorescence complementation (BiFC), to validate interactions. These methodologies are appropriate and consistent with those used in leading research on *T. gondii* virulence factors (32, 33). However, a more detailed analysis of the functional consequences of these interactions, such as their impact on host cell viability, immune evasion, or parasite replication, would strengthen the scientific merit. Additionally, integrating data from high-confidence protein-protein interaction mapping (34) could provide a more comprehensive view of GRA23′s role in host modulation.

In the GST pull-down screening for interacting proteins, the procurement of purified fusion proteins is fundamentally essential (35). In order to preserve the biological activity of these proteins, we specifically focus on obtaining soluble fusion proteins during the purification process, thus effectively reducing the incidence of false positives in our screenings. Through the systematic employment of GST pull-down and mass spectrometry methodologies, we utilized GRA23 as bait to identify interacting host proteins. Following stringent screening protocols, we successfully identified 35 candidate proteins that interact positively with GRA23. Subsequent GO and KEGG analyses prominently highlighted significant enrichment of these proteins in metabolic and immune-related pathways, such as dicarboxylate metabolism, primary bile acid biosynthesis, and the MAPK signaling pathway, thereby suggesting substantial shifts in *T. gondii*'s biosynthesis and metabolism subsequent to host cell invasion.

Notably, we confirmed the precise interactions between GRA23 and two candidate proteins, PEX3 and TRAP1, through the utilization of BiFC and co-IP methodologies. PEX3 and TRAP1, which are implicated in peroxisome biogenesis and modulation of host inflammatory responses, respectively, strongly suggest that GRA23 might strategically modulate peroxisomal functions through PEX3, thereby altering host metabolic pathways and potentially redirecting resources to support parasitic growth or undermining host defense mechanisms. Additionally, PEX3 play a critical role in lipid metabolism, and lipids are essential nutrients required for the proliferation of *T. gondii*. By affecting the function of PEX3, GRA23 may alter the lipid metabolism of host cells, providing more lipid resources to support the growth and reproduction of the parasite. These findings are aligned with recent discoveries in *T. gondii* secretory protein functions (36, 37). Furthermore, TRAP1, predominantly localized within mitochondria, functions as a crucial chaperone facilitating protein folding and protecting cells from stress-induced damage (38). Although this study identified the interaction between *T. gondii* GRA23 and host proteins, there are still some limitations. The research primarily relied on *in vitro* cell culture models, which cannot fully replicate the complex physiological environment of the host during natural infection. Additionally, *in vitro* experiments often use high-expression systems or artificially induced conditions, which may lead to protein overexpression or non-physiological localization, thereby affecting the authenticity and functional interpretation of the interactions. The observed GRA23-TRAP1 interaction therefore suggests that the parasite may advantageously leverage TRAP1′s chaperoning capabilities to stabilize its proteins within the host environment, potentially compromising cellular stress defense mechanisms and consequently increasing susceptibility to damage. Subsequent studies could involve constructing *T. gondii* strains with GRA23 gene knockout to observe changes in their proliferation, survival, and virulence within host cells. Additionally, analyzing the expression levels of peroxisomal and chaperone-related markers in infected animal tissues would help validate the functional significance of the GRA23-PEX3 and GRA23-TRAP1 interactions *in vivo*. In summary our study has successfully identified and rigorously validated interactions between GRA23 and host proteins PEX3 and TRAP1, thereby elucidating GRA23′s multifaceted role in key host cell processes such as reproduction, growth, and development. This research not only significantly deepens our understanding of GRA23′s molecular mechanisms but also provides critical insights and potential therapeutic targets for developing novel strategies against *T. gondii* infections, thus offering promising new avenues for its prevention and treatment.

## 5 Conclusions

This study identified and validated novel interactions between *T. gondii* GRA23 and host proteins PEX3 and TRAP1 through pull-down assays, mass spectrometry, BiFC, and Co-IP. The GRA23-PEX3 interaction suggests parasite modulation of peroxisomal functions, while GRA23-TRAP1 association indicates exploitation of host chaperone mechanisms. GO/KEGG analyses revealed significant enrichment in metabolic pathways and cellular processes related to reproduction, growth, and development. These findings provide the first evidence of GRA23′s direct interaction with host proteins, offering novel insights into *T. gondii* pathogenesis and identifying potential therapeutic targets for toxoplasmosis treatment in humans and animals.

## Data Availability

The original contributions presented in the study are included in the article/Supplementary material, further inquiries can be directed to the corresponding author.
